# Retraction: Eosin Y catalysed photoredox synthesis: a review

**DOI:** 10.1039/d2ra90108k

**Published:** 2022-11-07

**Authors:** Vishal Srivastava, Praveen P. Singh

**Affiliations:** Department of Chemistry, United College of Engineering & Management Naini Allahabad-211010 U.P. India; Department of Chemistry, United College of Engineering & Research Naini Allahabad-211010 U.P. India ppsingh23@gmail.com

## Abstract

Retraction of ‘Eosin Y catalysed photoredox synthesis: a review’ by Vishal Srivastava *et al.*, *RSC Adv.*, 2017, **7**, 31377–31392, https://doi.org/10.1039/C7RA05444K.

The Royal Society of Chemistry, with the agreement of the authors, hereby wholly retracts this *RSC Advances* review article due to significant portions of text overlap with a number of sources throughout the review article, in particular ref. 17, 24, 46, 49–54, 56–62 and 67 of the original article and ref. 1–6 below. Although many of these articles have been cited, it was not made clear that some of the text was reproduced from these articles.

Signed: Vishal Srivastava, Praveen P. Singh

Date: 13/10/2022

Retraction endorsed by Laura Fisher, Executive Editor, *RSC Advances*

## Supplementary Material

## References

[cit1] Romero N. A., Nicewicz D. A. (2016). Chem. Rev..

[cit2] Prasad Hari D., König B. (2014). Chem. Commun..

[cit3] Majek M., von Wangelin A. J. (2016). Acc. Chem. Res..

[cit4] Májek M. , Activation of arene-heteroatom bonds by photoredox catalysis with visible light, PhD Thesis, University of Regensburg, 2015, https://epub.uni-regensburg.de/34813/1/teza_server1.pdf

[cit5] Majek M., von Wangelin A. J. (2015). Angew. Chem., Int. Ed..

[cit6] Yadav A. K., Yadav L. D. S. (2014). Tetrahedron Lett..

